# Relationship between *Toxoplasma gondii* seropositivity and schizophrenia in the Lebanese population: potential implication of genetic polymorphism of *MMP-9*

**DOI:** 10.1186/s12888-020-02683-0

**Published:** 2020-05-27

**Authors:** Amata El Mouhawass, Amale Hammoud, Marouan Zoghbi, Souheil Hallit, Chadia Haddad, Kinda El Haddad, Saydeh El Khoury, Jennifer Tannous, Sahar Obeid, Mohamad Adnan Halabi, Nour Mammari

**Affiliations:** 1 Medical Laboratory Department, Holy Family University, Batroun, 5534 Lebanon; 2 Public Health Faculty, Jinan University, Tripoli, Lebanon; 3 Psychiatric Hospital of the Cross, Jal Eddib, 6096 Lebanon; 4 Faculty of Medicine, Saint-Joseph University, Beirut, Lebanon; 5 Faculty of Medicine and Medical Sciences, Holy Spirit University of Kaslik (USEK), Jounieh, Lebanon; 6 INSPECT-LB: Institut National de Santé Publique, Épidémiologie Clinique et Toxicologie, Beirut, Lebanon; 7 INSERM, Univ. Limoges, CH Esquirol Limoges, IRD, U1094 Tropical Neuroepidemiology, Institute of Epidemiology and Tropical Neurology, GEIST, Limoges, France; 8 Faculty of Arts and Sciences, Holy Spirit University of Kaslik (USEK), Jounieh, Lebanon

**Keywords:** Toxoplasmosis, Schizophrenia, *MMP-9* gene polymorphism, CNS

## Abstract

**Background:**

*Toxoplasma* multiplication and its persistence into the brain cause a local neuroinflammatory reaction, resulting synthesis of neurotransmitters involved in neurological disorders, especially schizophrenia. The Matrix metallopeptidase 9 (MMP-9) protein can play a major role in this neuroinflammation. It can promote extravasation and migration of infected immune cells into the brain. The objectives of this study are to determine the possible association between schizophrenia and toxoplasmosis and highlight the existence of gene polymorphism encoding MMP-9 protein’s in patients presented both schizophrenia and toxoplasmosis*.*

**Methods:**

A case-control study was conducted on 150 patients with schizophrenia (case group), and 150 healthy persons (control group). Groups were matched with age, gender, and place of residence. The survey was conducted using a questionnaire and a serological profile assay for specific IgG and IgM antibodies against *T. gondii*. Reverse transcription-polymerase chain reaction (RT-PCR) of gene polymorphism encoding MMP-9 was performed on 83 cases selected randomly.

**Results:**

Data show a significant association between toxoplasmosis (IgM+/IgG+ serological profile) and schizophrenia. Significant effects of raw meat consumption and contact with cats have been associated with the occurrence of schizophrenia. RT-PCR shows the presence of muted allele of *MMP-9* gene in selected cases whose present *T. gondii* serological profile IgM+/IgG+ and IgM−/IgG+ respectively.

**Conclusion:**

Toxoplasmosis may be one of the etiological causes of schizophrenia, and *MMP-9* gene polymorphism could be involved in the occurrence mechanism of this pathology following *Toxoplasma* infection.

## Background

*Toxoplasma gondii* (*T. gondii*) is an obligate intracellular protozoan parasite that can infect most warm-blooded animals, including humans. About one-third of the world’s population is infected with this parasite. After infection, the host usually maintains a chronic infection that is generally asymptomatic but can cause severe and potentially fatal disease in immunocompromised patients and fetuses [1]. When the infection occurs in pregnant women, contamination of the fetus is possible with different consequences depending on the stage of embryogenesis and the degree of maturity of the immune system, thus resulting in congenital toxoplasmosis [2]. The neurological disorders (hydrocephalus, microcephaly, mental retardation, intracranial calcification) or ocular (retinochoroiditis) of congenital toxoplasmosis reflect the neurological tropism of this parasite [3]. In the central nervous system (CNS), *T. gondii* infection is characterized by the differential detection of tachyzoites and tissue cysts in nerve cells. In response to this parasitic multiplication, a local neuroinflammatory reaction can be induced, resulting in brain damage and active synthesis of neurotransmitters that are involved in necrotizing brain lesions and neurological disorders occurrence [4]. In the brain, during the first stage of toxoplasmosis, studies show that astrocytes and microglia, are activated as immune cells [5, 6]. In immunocompromised patients, e.g., AIDS, or those receiving immunosuppressive therapy, reactivation of latent toxoplasmosis has occurred causing encephalic toxoplasmosis [7]. Recently, it has been shown that the latent toxoplasmosis, although often considered as asymptomatic and clinically unimportant, can modify host behavior in human and rodents. *Fabiani* et al.*,* demonstrated that this parasite could be an etiological factor for some neuropsychiatric diseases, especially Schizophrenia [8], and it has been described that this infection is a risk factor for the development of behavioral changes and other disorders such as Depression, Schizophrenia, Alzheimer and Parkinson’s diseases [9–12]. During an acute toxoplasmosis, the parasite is mainly present in peripheral tissues and blood; it has access to the brain via immune circulating cells [13–15]. In the brain, the balance between host immunity and defense mechanisms influence mostly the clinical manifestation of toxoplasmosis. These reactions could cause local neuroinflammation that has consistently been observed in both *T. gondii* infection and schizophrenia [16, 17]. Immune mediators released during this reaction can allow the occurrence of schizophrenia through their ability to modulate neurotransmission [18]. The mechanisms underlying these perturbations have been studied, and it was demonstrated that *T. gondii* has the ability to affect the synthesis of dopamine (DOPA), that is implicated in neurological and psychiatric disorders [19]. In Parkinson disease, *T. gondii* stimulates neurotransmitter pathways and promote degradation of DOPA-producing nerve cells [20].

In the CNS, tachyzoites Infected astroglia stimulate the production of Matrix Metallopeptidase-2 and -9 (MMP-2 and MMP-9) [21]. These secreted proteinases play a role in the migration of inflammatory cells at the sites of infection, representing a response that contributes to the fight against parasites and, thus, to neuroinflammation [22]. Previous studies have demonstrated that the activity of the MMP-9 protein is controlled by a functional 1562 C/T polymorphism in the promoter region of the human *MMP-9* gene. The T^− 1562^ allele has been shown to have a higher promoter activity in gene expression than the C-1562 allele [23]. The expression of this polymorphism could promote the invasion of immune cells infected by the *T. gondii*, which increase the parasite load in CNS. The increase of parasitic infection in the brain could stimulate nerve cells to produce neurotransmitters involved in the onset of schizophrenia. Two studies were performed, the first, to determine the possible association between schizophrenia and toxoplasmosis and the second, to highlight the existence of gene polymorphism encoding a proinflammatory protein MMP-9 in patients with schizophrenia and infected with *Toxoplasma*.

## Methods

### Studies designs

An observational epidemiological case-control study was conducted from February to September 2017, and a molecular epidemiological study was conducted from February to May 2018. In both studies, sampling was realized at the Psychiatric Hospital of the Cross, Lebanon. The case-control survey was conducted among 150 patients with a mean age of 56 years (SD = 12.92) (75 men and 75 women) randomly selected from a list of patients with schizophrenia admitted to the hospital for chronic management. Patients were diagnosed with schizophrenia by a psychiatrist at the hospital using the DSM-5 criteria. After eligibility criteria were determined, subjects were assigned identification numbers and randomized according to an online software, Research Randomizer (www.randomiser.org).

In parallel, the control group consists of 150 healthy volunteers selected randomly from the surrounding communities and matched by age, gender, and area of residence (rural or urban) with the cases average age of 58 years (SD = 27.99) (75 men and 75 women). Immunodeficient patients, pregnant women, and those who refused to give informed consent or to answer the questionnaire were excluded from the study.

### The population included in the molecular study

The study was conducted in 83 patients with schizophrenia selected randomly from the database of the case-control study using the same randomization technique.

### Information collection

The sociodemographic characteristics of patients (gender, age, nationality, residential area, education level, socioeconomic and marital status, employment, family history of schizophrenia, consumption of alcohol and drugs, and smoking and brain disease) were recorded from medical files. Each participant was asked to complete a questionnaire to obtain data on the usual consumption of raw meat, contact with cats and type of water drinking (tap water, well water and water Gallon). The consumption of raw meat and contact with cat were dichotomized into two groups (Yes/No). The type of water drinking were categorized into: tap, well and water gallon.

### Serological test for toxoplasmosis

A total of 5 ml blood sample from each participant was collected to perform the serologic analysis of toxoplasmosis. The serum was separated by centrifugation at 4000 rpm for 5 min. Antigen *Toxoplasma* enzyme-linked immunosorbent assay kits (Ag-Toxo ELISA kits) are used to measure IgG and IgM antibodies to *T. gondii* in the blood (Human, Wiesbaden, Germany). The results were quantified by calculating a ratio between the reactivity of the samples and a standard sample run on each microplate. The minimum detection sensitivity level of IgG or IgM antibodies to *Toxoplasma gondii* using Ag-Toxo ELISA or TOXO IgM u-capture ELISA kits is defined as a higher IgG or IgM ratio of *T. gondii* or equivalent to 1 international unit (IU) /ml. Both tests have a sensitivity of 100% and a specificity of 99%.

### TOXO IgG detection

Quantification of *Toxoplasma* IgG was performed by the Ag-Toxo ELISA kit for the detection of antibodies (Human, Wiesbaden, Germany). Microtiter wells as a solid phase are blankets of *Toxoplasma* antigens (Ag-TOXO) prepared from *T. gondii* parasites into which the serum is to be analyzed (the first phase). The anti-IgG conjugate (human anti-human IgG antibodies, labeled with peroxidase) is added. The latter attaches specifically to IgG antibodies. After washing tetramethylbenzidine (TMB) (or substrate) is added. TMB is a substrate-specific to the enzyme if the reaction is positive will be transformed and induce a blue color. A blue color appears it turns to yellow after stopping the enzymatic reaction. The intensity of the color is directly proportional to the concentration of Ac sought in the sample, therefore of *Toxoplasma* immunoglobulin G (TOXO IgG) Ac in the example.

### TOXO IgM detection

The Human TOXO IgM u-capture ELISA is intended for professional use (Human, Wiesbaden, Germany). It was used to assay IgM antibodies specific to *Toxoplasma*. A volume of 10 μl of each serum is added to the wells for the samples. After incubation, unbound serum components are removed by washing. During a second incubation step, *Toxoplasma* horseradish peroxidase (HRP) antigen is added to each well. It binds specifically to anti-*Toxoplasma* IgM antibodies. Finally, the TMB substrate is added. A blue color appears and turns yellow after stopping the reaction.

The intensity of the colors is directly proportional to the concentration of *Toxoplasma* IgM antibodies in the sample. The absorbance of the samples (case and control) is determined through an Elisa reader. The optical density (OD) is measured then calculated, taking into account the mean absorbance values of NC (negative control), CC (cut-off), PC (positive control).

The IgG and IgM assay are performed following an OD measurement in each well containing a sample (case or control). The OD measurement is performed using an ELISA reader (Human, Wiesbaden, Germany). The results are quantitative. It is carried out using a calibration curve consisting of the threshold control and the three positive controls. All assays are done in duplicate.

### DNA extraction

The DNA extraction was performed by the GenJET Whole Blood Genomic DNA Purification Mini Kit (Thermo Fisher Scientific, Waltham, MA USA). A volume of 200 μl of plasma was used for the extraction. The first step of lysis of the cells was carried out using Proteinase K and lysis solution. After 10 min of incubation at 56 °C, a DNA precipitation step was made by ethanol. Then two washes were successively carried out using wash buffer 1 and 2 alternated by centrifugation at 10000 rpm. Finally, DNA is eluted by 200 μl of elution buffer. Centrifugation was done at 10000 rpm and DNA obtained is stored at − 20 °C.

### *MMP-9* gene amplification

The amplification of the *MMP-9* gene was made using the Thermo Scientific Dream Taq Green PCR kit (Thermo Fisher Scientific, Waltham, MA USA). This kit contains a prepared Master Mix, which is a ready-to use solution containing DreamTaq DNA polymerase, optimized buffer, MgCl_2_, and dNTPs (deoxynucleotide triphosphates). The master mix is completed with two tracking dyes and a density reagent that allows direct loading of the PCR product onto a gel. The dyes in the master mix do not interfere with the performance of the PCR. It is capable of amplifying genomic DNA.

### PCR-RFLP (restriction fragment length polymorphism) “Identification of C1562T *MMP-9* Polymorphism”

The DNA extracted from each sample is amplified by the kit “Thermo Scientific Dream Taq Green PCR, Master Mix” following the procedures of the supplier (Thermo Fisher Scientific, Waltham, MA USA). A volume of 15 μl of each DNA sample is added to a PCR tube containing: 6 μl of DreamTaq Green PCR Master Mix (2X) and 2 μl of each primer (46.2 nmol) (Thermo Fisher Scientific, Waltham, MA USA).

forward primer is: 5′-GCCTGGCACATAGTAGGCCC-3 ‘.

reverse primer is: 5′-CTTCCTAGCCAGCCGGCATC-3 ‘.

The gene amplification is carried out following this program: Initial denaturation at 94 °C for 5 min, denaturation at 94 °C for 45 s, hybridization at 65 °C for 45 s, elongation at 72 °C for 45 s and elongation final at 72 °C for 7 min.

The 435 bp amplicon was cleaved at 244 bp and 191 bp with the restriction endonuclease Tru1I (MseI) ((Thermo Fisher Scientific, Waltham, MA USA). The enzymatic digestion was carried out according to the following Protocol, in each tube, 15 μl of PCR product was mixed with 13 μl of nuclease-free water, 2 μl of 10 × Buffer R and 1 μl of Tru1I enzyme. The whole was incubated at 65 °C for 3 h. After the necessary incubation period, the digestion is stopped at 80 °C for 30 min. For the disclosure of PCR products and enzyme digests, a 2% agarose gel was prepared to efficiently separate the amplified DNA fragments based on their charge and size.

### Statistical analysis

The Statistical Package for Social Sciences (SPSS) 25 was used for data analysis. The Student t-test was used to compare means for continuous variables in two groups. For categorical variables and to estimate the allele frequencies, the Chi-square (χ2) and Fisher tests were used. A backward multivariable logistic regression was performed, taking the presence vs. absence of schizophrenia as the dependent variable and taking all variables that showed a *p* < 0.1 in the bivariate analysis as independent variables. The Bonferroni correction compensates for that increase by testing each individual hypothesis at a significance level of α/m, where α is the desired overall alpha level and m is the number of hypotheses/tests conducted. Concerning this study we tested 15 variables in each model with a desired error of 0.05; therefore the Bonferroni correction would test each individual hypothesis at a *p* value of 0.05/15 = 0.003.

A *p* < 0.05 in the multivariable model was considered significant.

## Results

### Sociodemographic and other characteristics of the sample population

Details regarding sociodemographic and other characteristics associated with participants with or without schizophrenia are shown in Table 1. A significantly higher proportion of cases were single (89.3% vs 17.3%; *p* < 0.001), unemployed (62% vs 50%; *p* = 0.036), with a primary level of education (26% vs 42.7%; *p* = 0.007) compared to control group (Table 1). No significant difference was observed between the two groups regarding the socioeconomic level (Table 1).

**Table 1:** Sociodemographic and other characteristics of the population

		Patients without schizophrenia Frequency (%)	Patients with schizophrenia Frequency (%)	***p***-value
Marital status	Single	26 (17.3%)	134 (89.3%)	**< 0.001**
	Married	124 (82.7%)	16 (10.7%)	
Socioeconomic level^a^	No income	72 (48.0%)	85 (56.7%)	0.161
	Low	68 (45.3%)	54 (36.0%)	
	Intermediate	8 (5.3%)	11 (7.3%)	
	High	2 (1.3%)	0 (0.0%)	
Education level	Primary	64 (42.7%)	39 (26.0%)	**0.007**
	Complementary	55 (36.7%)	59 (39.3%)	
	Secondary	16 (10.7%)	32 (21.3%)	
	University	15 (10.0%)	20 (13.4%)	
Employment	Unemployed	75 (50.0%)	93 (62.0%)	**0.036**
	Employed	75 (50.0%)	57 (38.0%)	
		**Mean ± SD**	**Mean ± SD**	
Age		57.91 ± 27.99	55.91 ± 12.92	0.428

^a^Low USD < 1000; intermediate USD 1000–2000; and high USD > 2000; numbers in bold indicate significant *p*-values

### Effect of *Toxoplasma* risk factor and *T. gondii* infection on schizophrenia

The bivariate analysis, taking the absence/presence of schizophrenia as the dependent variable, showed that a significantly higher proportion of cases had a seropositive toxoplasmosis IgM/IgG compared to the control group (37.6% vs. 0.7%; *p* < 0.001) (Table 2). However, serological profile IgM−/IgG+ for *T. gondii* does not show a significant difference result (79.6% vs. 79.1%; 0.923) (Table 2). Besides, compared to the control group a significantly higher proportion of cases drank tap water (78% vs. 46%; *p* < 0.001) and had a continuous contact with cats (40.7% vs. 8%; p < 0.001). Also, results show that a higher proportion of cases group were smokers (60.7% vs. 22.7%; p < 0.001) and abusing illegal drugs (8% vs. 0%; p < 0.001) (Table 2). We could not detect any significant association between both groups for alcohol use disorder and brain disease (*p* > 0.05 for both variables) (Table 2).

**Table 2:** Bivariate analysis taking participants with or without schizophrenia as a dependent variable

		Patients without schizophrenia Frequency (%)	Patients with schizophrenia Frequency (%)	p-value
Toxoplasmosis IgM+/IgG+	Yes	1 (0.7%)	56 (37.6%)	**< 0.001**
	No	148 (99.3%)	93 (62.4%)	
Toxoplasmosis IgM−/IgG+	Yes	117 (79.1%)	74 (79.6%)	0.923
	No	31 (20.9%)	19 (20.4%)	
Presence of a cat at home	Yes	9 (6.0%)	55 (36.7%)	**< 0.001**
	No	141 (94.0%)	95 (63.3%)	
Contact with a cat	Yes	12 (8.0%)	61 (40.7%)	**< 0.001**
	No	138 (92.0%)	89 (59.3%)	
Water drinking	Tap	69 (46.0%)	117 (78.0%)	**< 0.001**
	Well	25 (16.7%)	8 (5.3%)	
	Water Gallon	56 (37.3%)	25 (16.7%)	
Consumption of raw meat	Yes	142 (94.7%)	140 (93.3%)	0.627
	No	8 (5.3%)	10 (6.7%)	
Smoking	Yes	34 (22.7%)	91 (60.7%)	**< 0.001**
	No	116 (77.3%)	59 (39.3%)	
Alcohol	Yes	10 (6.7%)	18 (12.0%)	0.112
	No	140 (93.3%)	132 (88.0%)	
Illegal drugs	Yes	0 (0.0%)	12 (8.0%)	**< 0.001**
	No	150 (100.0%)	138 (92.0%)	
Brain disease	Yes	1 (0.7%)	6 (4.0%)	0.056
	No	149 (99.3%)	144 (96.0%)	

Numbers in bold indicate significant *p*-values

### Variables affecting schizophrenia

A backward logistic regression, taking the absence/presence of schizophrenia as the dependent variable and the toxoplasmosis IgM+/IgG+ and IgM−/IgG+ as an independent variable, showed that being a smoker (ORa = 2.70), presence of a cat at home (ORa = 7.13) and having a seropositive IgM+/IgG+ profile (ORa = 34.55) would significantly increase the odds of being schizophrenic. However, being married (ORa = 0.04) and drinking from a gallon (Beta = 0.34) would significantly decrease the odds of schizophrenia (ORa = 0.040) (Table 3).

**Table 3:** Multivariate analysis taking the schizophrenia/control as a dependent variable

	OR	95% Confidence Interval		p-value
		Lower	Upper	
Marital status (married vs single^a^)	0.047	0.02	0.10	**< 0.001**
Smoking	2.70	1.24	5.88	**0.012**
IgG+/IgM+	34.55	4.01	297.09	**0.001**
Presence of a cat at home (Yes vs No^a^)	7.13	2.40	21.17	**< 0.001**
Water drinking (Gallon vs tap^a^)	0.34	0.14	0.83	**0.018**

Variable entered: Marital status, presence of a cat at home, contact with a cat at home, water drinking, smoking, IgG/IgM+, IgG+/IgM-, interaction between IgG/IgM+ and IgG+/IgM-

Hosmer and Lemeshow test was not significant (*p* = 0.894)

Nagelkerke R2 = 0.731 suggests that 73.1% of schizophrenic patients were explained by the independent variables included

^a^Reference group; numbers in bold indicate significant *p*-values

The interaction between IgG/IgM+ and IgG+/IgM- did not show any significant association with the presence of schizophrenia.

### Genetic polymorphism *MMP-91562 C > T* detection in schizophrenic patients

The 83 patients included in this study are divided into three groups: 40 individuals with a serology profile IgM+/IgG+ anti-*T. gondii*. Twenty-seven individuals with serological IgM−/IgG+ and 16 individuals with IgM−/IgG- serological profile.

To determine the presence or absence of the *MMP-9*1562 C > T polymorphism, the amplicon of the *MMP-9* gene was digested with the Tru1I enzyme (Msel). The revelation of the digested product showed a band of approximately 435 bp in patients with schizophrenia who have a serological profile of *T. gondii* IgM- and IgG-. The appearance of this band indicates the absence of digestion and, therefore, the absence of muted allele T in patients with schizophrenia who are not infected with *T. gondii* (Table 4). Statistically, 100% of included patients who do not present toxoplasmosis have 100% of wild type allele CC (*p* = 0.003). On the other hand, results showed the appearance of two bands of about 244 bp and another about 191 bp. These indicate the existence of the restriction site of the enzyme used and, therefore, the presence of the *MMP-9*1562 C > T gene polymorphism in patients with schizophrenia, who have a positive serological profile for *T. gondii*. 15% of included patients with serological *T. gondii* profile IgM+/IgG+ have homozygote wild type allele CC, 35% of them are heterozygote presented CT genotype and 50% of them are homozygote for muted allele TT (Table 4). While, 11.1% of included patients with serological *T. gondii* profile IgM−/IgG+ have homozygote wild type allele CC, 29.6% of them are heterozygote presented CT genotype, and 59.2% of them are homozygote for muted allele TT (Table 4). The revelation of muted allele T was significant in patients with toxoplasmosis (IgM+/IgG+ and IgM−/IgG+) compared to those who have not the infection (IgM−/IgG-) (67.5, 74, 0%; *p* < 0.001) (Table 4).

**Table 4:** Comparison of allelic and genotypic frequencies of the polymorphism *MMP-9*1562 C > T

	IgM+/IgG+ (***n*** = 40)	IgM−/IgG+ (***n*** = 27)	IgM−/IgG- (***n*** = 16)	p-value
MMP-91562 C > T				
CC	6 (15%)	3 (11.1%)	16 (100%)	**0.002**
CT	14 (35%)	8 (29.6%)	0 (0.0%)	**< 0.001**
TT	20 (50%)	16 (59.2%)	0 (0.0%)	**0.001**
Allele				
C	26 (32.5%)	14 (25.9%)	32 (100%)	**0.003**
T	54 (67.5%)	40 (74%)	0 (0.0%)	**< 0.001**

Numbers in bold indicate significant *p*-values

## Discussion

The conducted case-control study to determine a possible relationship between toxoplasmosis and schizophrenia in the Lebanese population demonstrates a significant association between schizophrenia and the positive profile of anti-*T. gondii* IgM/IgG. These results are confirmed by the multivariable analysis, which revealed a high odd ratio for cases with IgG+/IgM+ serology. This positive association is in correlation with the study of *Chen* et al. that identified a significant existence of anti-*T. gondii* IgG+ and IgM+ in patients with schizophrenia in the Chinese population [24].

Furthermore, *Abdollahian* et al. demonstrated a high prevalence of *T. gondii* infection in psychiatric patients with schizophrenia compared with the control group in the Iranian population [25], and a recent meta-analysis reported the association of toxoplasmosis reactivation with several psychiatric disorders, including schizophrenia [26]. Additional evidence has also reported that patients with a history of suicide, bipolar disorder, or major depression have a positive serologic profile for *T. gondii*-specific IgM antibodies [27]. Therefore, *T. gondii* has always been studied as an etiological factor affecting schizophrenia, depending on the region, tradition, climate, genetic factors, and many other factors, leading to the difference in studies; some studies proved a positive correlation and others found it negative. However, *Lindgren* et al. demonstrated a significant association between *T. gondii* seropositivity and psychotic manifestations [28]. A recent meta-analysis indicate that *Toxoplasma* seropositivity was primarily associated with schizophrenia and bipolar disease [29]. This evidence was already reported in other meta-analysis [26]. All studies supports the positive association of toxoplasmosis and the presence of schizophrenia.

In addition, the statistical analysis of this study revealed a significant effect of some interesting sociodemographic factors. Compared to the control group, the case group are mostly in contact with cats; they drink tap water and consume raw meat. These variables are essential risk factors for *T. gondii* seroconversion. Direct contact with infected cats is the primary cause of *T. gondii* seroconversion; Consumption of unclean water could cause toxoplasmosis via *T. gondii* oocysts; Ingestion of contaminated raw meat may cause infection with *T. gondii* cysts [30–32]. In addition to the risk factors that could explain the possible association of toxoplasmosis and schizophrenia, physiological mechanisms could strengthen this correlation by the fact that the presence of *Toxoplasma* in the CNS that certainly causes adequate tropism and deregulation of neurotransmitter gene expression, promoting the development of psychoses, including schizophrenia [24]. In the brain, *Toxoplasma* induce a high concentration of DOPA and tyrosine hydroxylase [33]. These data suggest that *T. gondii* could be an etiological factor for schizophrenia [34]. Clinically, toxoplasmosis and schizophrenia both induce similar alteration in brain morphology including pro-inflammatory immune cell infiltration and dendritic retraction in basolateral amygdala accompanied by reduced corticosterone secretion [8, 35, 36]. Thus, the relationship between chronic or acute *Toxoplasma* infection and schizophrenia seems to exist. Moreover, toxoplasmosis has already been demonstrated that it is associated with depression [37] [9, 38] and Parkinson’s disease occurrence [20, 39] and it remains that this infection is mainly persist in schizophrenic patients, associated with depressive symptoms and high-grade inflammation [40]. To understand the mechanism by which toxoplasmosis could be involved in the onset of schizophrenia, several studies have been carried out on the expression of pro-inflammatory proteins activated during this infection. In this study we suggested the implication of MMP-9 protein in the occurrence of schizophrenia following *Toxoplasma* infection. MMP-9 is part of MMPs family that involve in several neurological pathogenesis including parasitic infections, such as, protozoa and helminths. During these infections, MMPs could disrupt the blood-brain barrier by promoting infiltration of leukocytes to maintain the survival of neural cells [41]. During cerebral toxoplasmosis, MMP-9 plays a role in the degradation of the extracellular matrix components and in the extravasation of leukocytes in the inflammation sites [22, 23]. In addition, the experimentation have been shown that The *MMP-9* gene is expressed by neurons and adult glial cells in the brain. The encoded protein of this gene is released in response to the increased neuronal activity under physiological and pathological conditions [42] and, In murine model, the neuroinflammation-induced by oxidative stress is mediated by MMP-9 through microglia activation. Authors have suggested that this metalloprotein lead to the pro-inflammatory cytokine secretion and microglia activation in early psychosis patients including those with schizophrenia [43]. Convincing evidence suggested that the MMP-9 protein could contribute to the onset of neurological disorders, confirmed by serum MMP-9 level that was significantly higher in schizophrenic patients [44].

Although the implication of MMP-9 in schizophrenia onset was studied, possible implication of *MMP-9*1562 C/T gene polymorphism is still to be clarified. Results of this study show the presence of the *MMP-9*1562 C/T gene polymorphism in schizophrenic patients with toxoplasmosis. The absence of the polymorphism has been reported in patients with schizophrenia who do not have toxoplasmosis. Studies have reported that the 1562C/T polymorphic regulatory site of the *MMP-9* gene influences the rate of transcription and translation of the MMP-9 protein, which will result in overexpression of the latter. The mutated (polymorphic) allele significantly seems positive in schizophrenic patients [45] and other bipolar disease [46–48]. Although the etiology of schizophrenia seems to involve interactions between many genes, some of which code for proteins that regulate synaptic plasticity, notably the *MMP-9* gene that has been widely studied in psychiatric disorders related to schizophrenia [42, 45], it should be acknowledged that polymorphism of complex interplay of the *MMP-9* gene with the other genes and environmental risk factors could mediate cerebral toxoplasmosis, which contributes to a better explanation of the occurrence of schizophrenia.

Some limitations are identified in this study, we note that this study was based on a Lebanese population. However, according to our knowledge, to date, this is the first study evaluating the association of *T. gondii* seroprevalence and schizophrenia in a case-control study of the Lebanese population. Nevertheless, it would be interesting to carry out another study based on a larger population to define better the occurrence of schizophrenia correlated to the seropositive status of toxoplasmosis. In the molecular study, small sample size and the lack of a control group were noted it would, therefore, be interesting to do the case-control study to confirm the association of the polymorphism of the *MMP-9* gene with *T. gondii* infection and the occurrence of schizophrenia.

## Conclusion

*T. gondii* is an intracellular parasite that has a tropism for the CNS. Although it is generally asymptomatic in immunocompetent hosts, it can lead to severe psychiatric, neurological disorders. Several studies have shown that *T. gondii* affects the synthesis of neurotransmitters, particularly DOPA, in infected individuals, which could lead to personality changes, psychotic symptoms, and in some cases, neurological and psychiatric disorders. The results of this study show that *T. gondii* appears to be an etiological factor of schizophrenia.

The MMP-9 protein is known by its important implication in the dissemination of *T. gondii* in the CNS. It appears to be involved in one of the etiological mechanisms of cerebral toxoplasmosis associated with the onset of schizophrenia. The polymorphisms of the *MMP-9* gene have been implicated in the development of neurological disorders. The results show that the 1562 C > T polymorphism is present in patients with schizophrenia who have a positive anti-*T. gondii* serological profile.

The result of this study leads us to another alternative way of controlling the onset of schizophrenia. Awareness and education of vulnerable persons regarding hygiene and eating habits could be a means of protection against *Toxoplasma* infection and subsequently reducing the incidence of schizophrenia.

## Data Availability

Authors do not have the right to publicly share information about the data in accordance with the policies and requirements of their institutions. However, they can share the data following a request addressed to the corresponding author.

## Abbreviations

Ac: Antibody
Ag-Toxo ELISA: Antigen *Toxoplasma* Enzyme-Linked Immunosorbent Assay
bp: base pair
C: Cytosine
CC: Cut-off
CNS: Central Nervous System
DNA: DeoxyriboNucleic Acid
dNTPs: DeoxyNucleotide TriPhosphates
DOPA: Dopamine
HRP: HoRseradish Peroxidase
Ig: Immunoglobulin
IU: International Unit
MMP-2: Metallopeptidase-2
MMP-9: Matrix metallopeptidase − 9
NC: Negative Control
OD: Optical Density
OR: Odd Ratio
PC: Positive Control
PCR-RFLP: Polymerase Chain Reaction Restriction Fragment Length Polymorphism
RT-PCR: Reverse transcription-polymerase chain reaction
SPSS: Statistical Package for Social Sciences
T: Thymine
*T. gondii*: *Toxoplasma gondii*
TMB: TetraMethylBenzidine
μl: microliter
χ2: Chi-square
