# Correction to: Relationship between Toxoplasma gondii seropositivity and schizophrenia in the Lebanese population: potential implication of genetic polymorphism of MMP-9

**DOI:** 10.1186/s12888-020-02727-5

**Published:** 2020-06-23

**Authors:** Amata El Mouhawess, Amal Hammoud, Marouan Zoghbi, Souheil Hallit, Chadia Haddad, Kinda El Haddad, Saydeh El Khoury, Jennifer Tannous, Sahar Obeid, Mohamad Adnan Halabi, Nour Mammari

**Affiliations:** 1 Medical Laboratory Department, Holy Family University, Batroun, 5534 Lebanon; 2 Public Health Faculty, Jinan University, Tripoli, Lebanon; 3 Psychiatric Hospital of the Cross, Jal Eddib, 6096 Lebanon; 4 Faculty of Medicine, Saint-Joseph University, Beirut, Lebanon; 5 Faculty of Medicine and Medical Sciences, Holy Spirit University of Kaslik (USEK), Jounieh, Lebanon; 6 INSPECT-LB: Institut National de Santé Publique, Épidémiologie Clinique et Toxicologie, Beirut, Lebanon; 7 INSERM, Univ. Limoges, CH Esquirol Limoges, IRD, U1094 Tropical Neuroepidemiology, Institute of Epidemiology and Tropical Neurology, GEIST, Limoges, France; 8 Faculty of Arts and Sciences, Holy Spirit University of Kaslik (USEK), Jounieh, Lebanon

**Correction to: BMC Psychiatry (2020) 20:264**

**https://doi.org/10.1186/s12888-020-02683-0**

Following publication of the original article [1], the authors identified multiple errors in the original article;

1. The Methods and Results section contained incorrect sentences, and the changes have been highlighted below in bold typeface.

Methods:

**Polymerase Chain Reaction-Restriction Fragment Length Polymorphism (PCR-RFLP) of** gene polymorphism encoding MMP-9 was performed on 83 cases selected randomly.

Results:

**PCR-RFLP** shows the presence of muted allele of MMP-9 gene in selected cases whose present T. gondii serological profile IgM+/IgG+ and IgM-/IgG+ respectively.

2. The co-author’s names Amata El Mouhawess and Amal Hammoud possessed minor typos, and the names have been corrected.

Incorrect names:

Amata El Mouhawass

Amale Hammoud

Correct names:

Amata El Mouhawess

Amal Hammoud

The author group has been updated above and the original article [1] has been corrected.
